# Mesoporous Silica Nanoparticles: Types, Synthesis, Role in the Treatment of Alzheimer’s Disease, and Other Applications

**DOI:** 10.3390/pharmaceutics15122666

**Published:** 2023-11-24

**Authors:** Bhagavathi Sundaram Sivamaruthi, Devesh U. Kapoor, Rajiv R. Kukkar, Mansi Gaur, Gehan M. Elossaily, Bhupendra G. Prajapati, Chaiyavat Chaiyasut

**Affiliations:** 1 Office of Research Administration, Chiang Mai University, Chiang Mai 50200, Thailand; sivamaruthi.b@cmu.ac.th; 2 Innovation Center for Holistic Health, Nutraceuticals, and Cosmeceuticals, Faculty of Pharmacy, Chiang Mai University, Chiang Mai 50200, Thailand; 3 Department of Pharmacy, Dr. Dayaram Patel Pharmacy College, Bardoli 394601, Gujarat, India; dev7200@gmail.com; 4 School of Pharmacy, Raffles University, Neemrana 301705, Rajasthan, India; 5 Rajasthan Pharmacy College, Rajasthan University of Health Sciences, Jaipur 302033, Rajasthan, India; 6 Department of Basic Medical Sciences, College of Medicine, AlMaarefa University, P.O. Box 71666, Riyadh 11597, Saudi Arabia; jabdelmenam@um.edu.sa; 7 Shree S. K. Patel College of Pharmaceutical Education and Research, Ganpat University, Mehsana 384012, Gujarat, India

**Keywords:** silica nanoparticles, Alzheimer’s disease, drug delivery, brain targeting, theranostic

## Abstract

Globally, many individuals struggle with Alzheimer’s disease (AD), an unrelenting and incapacitating neurodegenerative condition. Despite notable research endeavors, effective remedies for AD remain constrained, prompting the exploration of innovative therapeutic avenues. Within this context, silica-based nanoplatforms have emerged with pronounced potential due to their unique attributes like expansive surface area, customizable pore dimensions, and compatibility with living systems. These nanoplatforms hold promise as prospective interventions for AD. This assessment provides a comprehensive overview encompassing various forms of mesoporous silica nanoparticles (MSNs), techniques for formulation, and their applications in biomedicine. A significant feature lies in their ability to precisely guide and control the transport of therapeutic agents to the brain, facilitated by the adaptability of these nanoplatforms as drug carriers. Their utility as tools for early detection and monitoring of AD is investigated. Challenges and prospects associated with harnessing MSNs are studied, underscoring the imperative of stringent safety evaluations and optimization of how they interact with the body. Additionally, the incorporation of multifunctional attributes like imaging and targeting components is emphasized to enhance their efficacy within the intricate milieu of AD. As the battle against the profound repercussions of AD persists, MSNs emerge as a promising avenue with the potential to propel the development of viable therapeutic interventions.

## 1. Introduction

**Figure 1:**
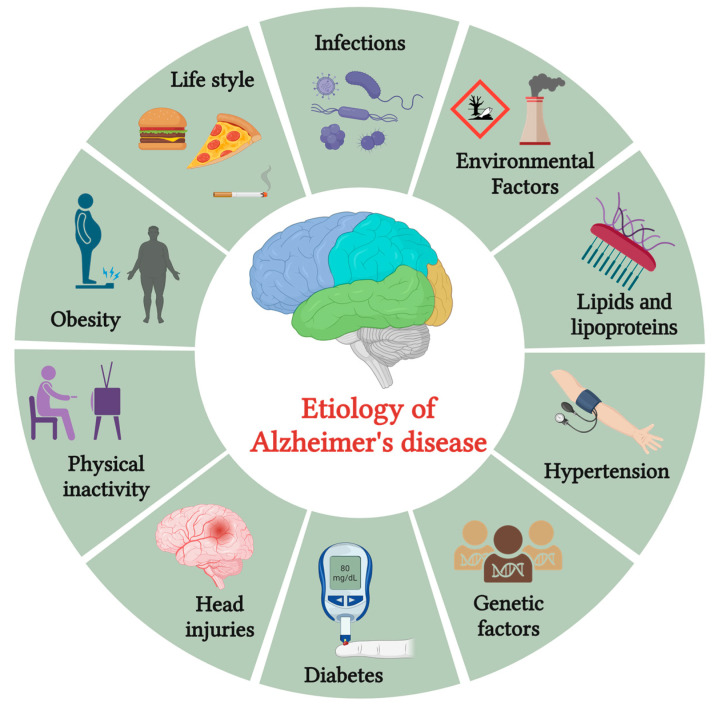
The etiology of Alzheimer’s disease (created using BioRender.com).

Alzheimer’s disease (AD) is the most common type of dementia, which takes its name from the German psychiatrist Alois Alzheimer. It is a slow-progressing neurodegenerative condition that affects the brain, leading to cognitive decline and memory loss. The hallmark features of AD include the presence of neuritic (also known as senile) plaques and neurofibrillary tangles. Alois Alzheimer, a pioneering German neurologist, made a significant observation while studying the brain of his first patient, who exhibited symptoms of memory loss and personality changes before passing away. Upon examination, Alzheimer noticed the presence of abnormal protein deposits known as amyloid plaques and a substantial loss of nerve cells (neurons) in the patient’s cerebral cortex, the brain’s outer layer responsible for complex cognitive functions [1]. Emil Kraepelin is credited with coining the term “Alzheimer’s disease” for the first time in his textbook on psychiatry [2]. AD affects approximately fifty million individuals worldwide, expected to double every 5 years. By 2050, it is projected to reach a staggering 152 million cases. Its far-reaching effects extend beyond individual suffering, affecting emotional, financial, and societal aspects. The assessed annual global cost of AD is about USD 1 trillion. This widespread prevalence and economic burden highlight the urgent need for effective treatments and interventions to alleviate the suffering caused by this devastating neurodegenerative disorder [3,4]. AD is a complex and multifactorial condition influenced by various risk factors depicted in Figure 1.

These factors encompass various contributors, including advancing age, genetic predisposition, vascular disorders, infections, and environmental influences such as exposure to heavy metals or trace elements. As individuals age, the risk of developing AD increases, making age a significant risk factor. Genetic factors play a crucial role, with certain gene variants associated with a higher susceptibility to the disease. Infections and inflammation have been implicated as potential triggers, with chronic infections or immune responses potentially influencing AD development.

As we age, cognitive decline can result from several factors, including cerebral disorders like AD, non-neurological causes like intoxications, circulatory issues affecting brain oxygen supply, and nutritional deficiencies (e.g., lack of vitamin B_12_). Additionally, medical conditions such as tumors may also impact cognitive abilities. Taking care of our brain health through regular exercise, a balanced diet, and mental stimulation is crucial to maintaining cognitive function as we age [5,6]. AD causes cognitive impairment and memory loss due to abnormal protein deposits called neurotic plaques and neurofibrillary tangles (NFTs) in the brain. Although extensively studied, the exact reasons behind these changes, such as amyloid-beta (Aβ) plaques and NFTs formation, remain unclear. Researchers continue their efforts to fully comprehend the precise mechanism triggering these brain changes, which is essential for developing effective treatments for AD [7,8].

Several medications (approved by regulatory agencies, including the U.S. Food and Drug Administration (FDA) and the European Medicines Agency (EMA)) can help to tackle symptoms and diminish the progression of AD for some individuals [9]. Cholinesterase inhibitors such as donepezil, rivastigmine, galantamine, and N-methyl-D-aspartate (NMDA) receptor antagonists, including memantine, are the most employed therapeutics for AD management [10]. Nanomedicine has revolutionized the administration of diagnostics and medications, leading to remarkable AD detection and treatment enhancements.

Hasan et al. (2023) extensively examined many nanomedicines that can scavenge reactive oxygen species (ROS), effectively mitigating oxidative stress in AD [11]. Liu et al. (2022) comprehensively explored several small molecular fluorescent probes with favorable characteristics, including water solubility, blood–brain barrier (BBB) permeability, and adaptability for fine-tuning photophysical and biological attributes. Their research focuses on advancing the field of AD diagnosis by developing these versatile probes [12].

Researchers have explored innovative nanomedicines encapsulated with liposomes, solid lipid nanoparticles, and polymeric micelles to revolutionize drug delivery. These tiny structures hold the potential to target diseases to enhance precision. MSNs exhibit noteworthy potential against AD due to their exceptional attributes like stable physiochemistry, customizable pore size and volume, significant surface area, and simple adaptability through functionalization [13,14]. MSNs showed exceptional biological properties, including low toxicity, good biocompatibility, and biodegradability [13,15,16]. The objective of the present manuscript was to summarize the role of mesoporous silica nanoparticles in the treatment of AD and other applications. Mendiratta et al. (2019) explored the multifaceted applications of MSNs in brain regeneration and cancer therapy, including their role in overcoming the BBB for treatment, and they discussed a single type of MSNs [17].

In the present review, we have discussed the different types of MSNs, including physical, chemical, and biological stimuli-responsive MSNs. Besides their role in AD management, the application of MSNs in wound healing and tissue regeneration and their biocatalyst properties were also discussed.

## 2. Pathology of AD

**Figure 2:**
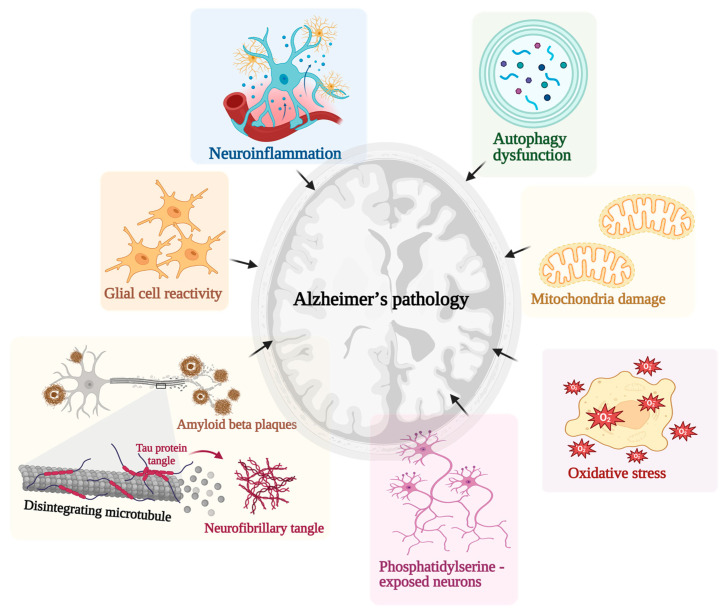
The major pathological influences of Alzheimer’s disease (created using BioRender.com).

AD is a slow and progressive neurodegenerative condition that severely impairs memory and cognitive function. AD is one of the major causes of dementia in the elderly, presenting significant challenges to public health and affecting countless individuals and their families worldwide [18]. The major pathological influences of AD are represented in Figure 2.

Briefly, in AD, the loss of neurons is a fundamental consequence. This process starts with the buildup of Aβ plaques and NFTs, triggering a chain of events that disrupts synaptic function and eventually leads to neuronal death. Initially, this disruption affects communication between neurons, resulting in cognitive deficits. As the disease progresses, neurons may die due to excitotoxicity from excessive glutamate release or undergo apoptosis. Brain regions critical for memory (hippocampus and cerebral cortex) are particularly susceptible to this neuronal loss, contributing to the hallmark memory impairment and cognitive decline seen in AD patients [19].

In AD, inflammation also plays a significant role. The brain’s immune response is activated when abnormal protein plaques (like Aβ) and NFTs build up. Microglia, the brain’s resident immune cells, try to clear these proteins, but their response becomes imbalanced in AD, leading to chronic neuroinflammation. This ongoing inflammation releases harmful substances like pro-inflammatory cytokines and reactive oxygen species (ROS), damaging neurons and accelerating the disease’s progression. Understanding and managing this inflammation is crucial for potential treatments, as it could help slow down the neurodegenerative process and reduce neuronal damage in AD [20]. Aggregating Aβ plaques alongside tau proteins can potentially escalate the production of detrimental ROS, thereby initiating a state of oxidative stress. In a subsequent cascade, this oxidative stress inflicts harm upon neurons, incites inflammatory responses, and plays a role in the progression of apoptosis, thereby exacerbating the severity of the ailment. Delving deeper into these variables could potentially open avenues for novel therapeutic interventions and enhance support for individuals grappling with AD [21].

AD involves a pivotal facet of the emergence of NFTs deep within neurons. These NFTs consist of hyperphosphorylated tau protein, which plays a crucial role in upholding the stability of neuronal structures. AD, an aberrant process, unfolds, inducing hyperphosphorylation of tau proteins [22]. This abnormal modification causes these proteins to detach from their customary positions along microtubules, subsequently amassing into NFTs. This disrupts neurons’ usual function and conveyance, culminating in neuronal loss. The distribution pattern of these NFTs adheres to a specific sequence, originating in the entorhinal cortex and gradually disseminating to other cerebral regions in tandem with the disease’s progression. This tau-centric mechanism is significant in AD, contributing to cognitive function and neuronal count decline. A comprehensive comprehension of this intricate process forms the bedrock for conceiving prospective therapeutic interventions that either decelerate or arrest the disease’s advancement [23].

Concurrent with this, perturbations in mitochondrial function interlock with AD pathology. Mitochondria, the powerhouses of cells, are indispensable in safeguarding neuronal well-being. In AD, disrupted mitochondrial function leads to compromised energy generation and an augmented susceptibility to oxidative stress. These factors synergistically advance neuronal degeneration [24,25].

## 3. Current Treatments and Their Limitations

Two primary categories of medications are employed to address AD. Cholinesterase inhibitors, namely galantamine, donepezil, and rivastigmine, are designated for employment in cases ranging from mild to severe AD and Parkinson’s. Conversely, Memantine operates as a dopamine agonist, influencing neurotransmitter operations within the brain. Its objective lies in enhancing cognitive capabilities in individuals grappling with AD. Nevertheless, it is prudent to acknowledge that while these pharmacological interventions can offer relief, their capacity might fall short of comprehensively arresting or reversing the disease’s advancement [26].

The medications (like Acetylcholinesterase inhibitors; AChEIs) used for AD could cause side effects, and they primarily affect the gastrointestinal tract, causing diarrhea, nausea, and vomiting. Taking the medication after a meal in the morning can help reduce these effects [27]. If administered through a transdermal patch, like rivastigmine, there is a possibility of developing a rash at the application site. About 5 to 20% of AD patients are affected by side effects, which are generally mild and temporary [28]. However, caution is necessary with AChEIs, as they can lead to bradycardia (slow heart rate) and an increased risk of fainting.

For this reason, individuals with severe cardiac arrhythmias, especially bradycardia or syncope, should avoid these medications. Also, patients with a history of active peptic ulcers, gastrointestinal bleeding, or uncontrolled seizures should avoid AChEIs. Consulting a healthcare professional before starting or stopping any medication is vital to ensure safety and effectiveness, tailoring treatment to individual health needs [29].

Memantine stands out as a distinct medication for AD treatment, which functions by blocking NMDA receptors related to glutamate signaling in the brain [30]. Memantine is eliminated mainly through the kidneys and remains in the system for approximately 70 h [31]. The FDA has approved memantine to treat moderate and severe AD alone or combined with AChEIs. Studies indicated that memantine alone can benefit patients with moderate to severe AD, enhancing daily activities and cognitive function [32]. An advantage of memantine is its compatibility with AChEIs, as they work differently and complement each other’s effects without increasing side effects. For those with moderate or advanced dementia, considering treatment duration and dosage is vital. This approach has shown positive outcomes, improving overall function and the condition of AD patients. Consulting healthcare professionals is crucial to creating individualized treatment plans for optimal care [33].

In addition to traditional pharmacologic therapies, some patients opt for alternative treatments like huperzine A, a nutraceutical. Huperzine A showed a beneficial effect on AD patients’ memory function and daily activities [34]. Studies have indicated that vitamin D deficiency increases the risk of dementia, irrespective of its underlying cause [35]. Therefore, vitamin D supplementation is suggested for individuals diagnosed with deficiency to potentially mitigate the risk of dementia. As of September 2021, it is important to note that DMARDs (disease-modifying anti-rheumatic drugs) and immunotherapy are not conventional treatments for AD. DMARDs are utilized to manage autoimmune and inflammatory conditions like rheumatoid arthritis. Conversely, immunotherapy involves treatments that target the immune system to address specific diseases, such as cancer or certain autoimmune disorders. No approved DMARDs or immunotherapies are specifically designated to treat AD [1].

## 4. Mesoporous Silica Nanoparticles (MSNs)

A wide array of nanomaterials, such as liposomes, dendrimers, carbon nanotubes, gold and iron oxide nanoparticles, titanium dioxide, and MSNs, are employed in various applications. These nanomaterials offer unique properties that enable tailored functionalities in fields like drug delivery, imaging, and environmental remediation [36,37]. Among the various nano-systems, MSNs are one of the useful materials with many mesopores. This unique configuration of MSNs holds several advantages, including significant surface area, large pore volume, pore diameter, chemical and thermal stability, biocompatibility, and biodegradability. The exceptional properties of MSNs make them an attractive candidate for diverse fields, including drug delivery, catalysis, and biomedical applications [38,39].

The evolution of MSNs can be described over three generations. The first generation comprised pioneering MSNs like MCM-41 and SBA-15, demonstrating mesoporous materials. The second generation involved the development of MSNs with nanosized pores and adjustable compositions, setting benchmark in vitro and in vivo evaluations. Finally, the third generation represents multifunctional MSNs, where nanoparticles have multiple functionalities for enhanced performance in various applications. This progressive advancement in MSNs showcases the continuous refinement and diversification of these nanoparticles, expanding their potential in diverse fields of research and technology [40].

### 4.1. Types of MSNs

**Figure 3:**
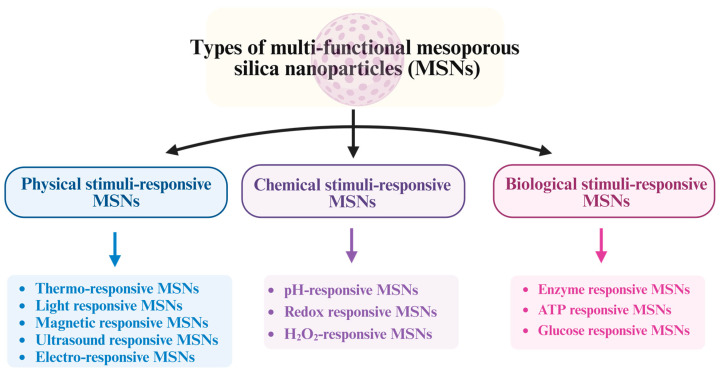
Different types of MSNs (created using BioRender.com).

MSNs have been classified according to their surface area, pore size, particle size, and preparation method (Figure 3).

#### 4.1.1. Physical Stimuli-Responsive MSNs

Temperature is a widely employed and straightforward stimulus that effectively controls the response of MSNs delivery systems in various biomedical applications. Temperature-sensitive MSNs delivery systems are engineered to maintain an inactive state at the body’s physiological temperature (37 °C) to ensure safe circulation within the body. This design allows them to remain stable and inert until they reach the target site, where a temperature change triggers drug release for precise and controlled therapeutic delivery. Upon reaching tumor-targeted sites, these delivery systems can be activated at higher temperatures, particularly when combined with hyperthermia therapy, enabling controlled drug release. This activation is triggered by the conformational change of conjugated polymeric ligands [41]. Poly (N-isopropyl acrylamide) (PNIPAM) is one of the widely recognized and extensively used temperature-sensitive polymers in scientific research [42]. Its conjugation to MSNs has been explored to enhance the thermo-responsive characteristics of the nanoplatforms. PNIPAM-conjugated MSNs hold immense potential in biomedical applications where they can respond to localized hyperthermia conditions or other temperature changes within a tumor microenvironment [43].

Thermo-responsive MSNs: Thermo-responsive systems, also known as temperature-sensitive delivery systems, are an extensively studied group of physical stimuli-responsive technology. These systems can respond to changes in temperature, allowing for controlled drug release and targeted therapies in various biomedical applications. These systems can be customized to function effectively within the body’s typical temperature range while also exhibiting sensitivity to specific tumor-targeted temperatures, like hyperthermia (42 °C). This capability holds significant potential for developing targeted therapies, providing a promising approach to treating various medical conditions [44,45].

Researchers have explored hybrid nanocarriers, combining MSNs with supported lipid bilayers (SLB), as an innovative drug delivery system. Zhang and his colleagues successfully created biocompatible SLB-MSN with high drug loading capacity. Following the synthesis of MSNs through the sol-gel method, a temperature-responsive SLB was applied to the MSNs using sonication to seal the mesopores fully. This step ensures controlled drug release and enhances nanoparticle stability and functionality. The effective coating of MSNs with SLB forms spherical nanoscale particles. Notably, the release test demonstrated a significant 30–40% increase in drug release at 47 °C compared to 37 °C, indicating the temperature-sensitive characteristics of the coated nanoparticles. This temperature-responsive behavior is attributed to the lipid bilayer’s transition temperature (Tm), enhancing permeability beyond this critical point [46]. Among physical stimuli, thermo-responsive strategies are commonly used. However, there are concerns about ensuring the safety and sensitivity of novel thermo-responsive nanomaterials for effective functionality in a physiological environment.

Light-responsive MSNs: Light has emerged as an appealing stimulus for precise and controlled drug release, offering remote activation with high spatial and temporal accuracy. Its versatile control parameters, including wavelength, intensity, exposure duration, and beam size, make it a promising tool for applications related to targeted drug delivery. In recent times, a notable surge of interest has emerged in the realm of advancing light-responsive systems. Many of these innovative systems leverage light-sensitive chromophores such as azobenzene and spiropyran. These chromophores enable precise and regulated reactions to light-based stimuli [47,48,49]. Wu et al. (2023) developed an inventive light-responsive drug carrier, β-CD-MSN. This carrier’s characterization involved various techniques, including SEM, FTIR, UV, BET, XRD, and TGA. To probe the loading and subsequent release capabilities of β-CD-MSN, quercetin (QCT) was chosen as a representative drug surrogate. In vitro drug release testing demonstrated that QCT-β-CD-MSN exhibited favorable characteristics, with slow and light-controlled drug release capabilities. The CCK8 tests revealed that β-CD-MSN effectively reduced the toxicity of QCT, showcasing the potential of this light-responsive drug delivery system for promising applications [47].

Yang et al. (2022) engineered a red light-responsive, self-destructive carrier by incorporating PEG modification and diselenide-bridged MSNs. This innovative carrier system shows promising potential for targeted drug delivery and can release therapeutic agents upon exposure to red light. The carrier is designed to carry the chemotherapy drug doxorubicin and the photosensitizer methylene blue, enabling a combination of chemo-photodynamic therapy for enhanced effectiveness. During photodynamic therapy (PDT) with low-dose red light irradiation, ROS triggers the cleavage of diselenide bonds, leading to the degradation of the organosilica matrix and facilitating the simultaneous release of two drugs. This process enhances the therapeutic efficacy of drug delivery and precision for effective treatment outcomes [48].

Magnetic responsive MSNs: Innovative magnetically guided and responsive delivery strategies are promising in enhancing drug and biologically active molecule therapies. These approaches enable targeted distribution of carriers to the desired site, controlled release, and minimized off-target interactions, leading to improved therapeutic profiles and treatment efficacy [49]. Jia et al. (2020) successfully synthesized Fe_3_O_4_ nanoparticles with precise size and uniformity, followed by a coating process involving mesoporous silica and polydopamine to enhance their properties. The distinctive core-shell architecture enhances the drug loading capacity and photothermal conversion efficiency of magnetic nanomaterials, highlighting the promising potential for advanced therapeutic applications. Polydopamine’s reducibility enables the conversion of Fe^3+^ to Fe^2+^, triggering the generation of hydroxyl radicals through the Fenton reaction, effectively inducing tumor cell death. Magnetic nanomaterials offer the potential for a dual approach, combining photothermal and chemodynamic therapies, to target and treat tumors in the future. This integrated strategy holds promise in advancing cancer treatment with enhanced precision and effectiveness [50].

Ultrasound responsive MSNs: Ultrasound refers to pressure waves in a medium with frequencies below 20,000 Hz, which are too low for human hearing perception. Ultrasound’s favorable properties, like low absorption by water and tissue, enable non-invasive imaging with deep penetration and controllable frequencies. These advantages enhance its potential for drug delivery applications [51]. Du et al. (2020) introduced a pioneering approach by merging magnetic mesoporous silica nanoparticles with microbubbles (MAG-MSN-MBs). This innovative system enables ultrasound-mediated imaging and gene transfection, offering promising applications in biomedical research and therapies and opening new possibilities in medical applications. Plasmid DNA (pDNA) is efficiently encapsulated inside the pores of MAG-MSN. These pDNA-loaded MAG-MSN were subsequently incorporated into lipid microbubbles, creating a promising platform for gene delivery applications. The gene vector exhibited outstanding biocompatibility, maintaining stable DNA binding while displaying efficient ultrasound imaging capabilities and responsive magnetic properties. It is a promising and versatile tool for various biomedical applications. The introduction of polyethyleneimine (PEI) modification to MAG-MSN displayed effective protection of the loaded pDNA from enzyme degradation, ensuring its stability and effectiveness in gene delivery applications. Encapsulating M-MSNs in lipid microbubbles significantly reduced their cytotoxicity. A magnetic field attracted MAG-MSN-MBs to the tumor area, demonstrating their potential as targeted drug delivery carriers. Ultrasound-targeted microbubble destruction (UTMD) is a novel technique that enhances pDNA delivery efficiency by releasing loaded MAG-MSN and promoting their delivery to tumor tissue. UTMD opens the blood–tumor barrier, increases cytomembrane permeability, and improves the overall therapeutic efficacy of the treatment [52].

Electro-responsive MSNs: Electric fields have proven effective in triggering controlled drug release either in pulsed or sustained manners, owing to their precise dosage control. Various complementary strategies are often employed to optimize drug loading in electro-responsive systems [53]. Zhao’s research group designed a surface coating for titanium implants by incorporating ibuprofen (IBP)-loaded MSN into a chitosan hydrogel to enhance the implant’s performance and improve post-operative recovery and tissue integration. Chitosan hydrogel served as an efficient sealing agent, enabling a controlled and sustained release of the encapsulated drug. The process involved dispersing IBP-loaded MSNs in a chitosan solution, creating a chitosan/MSN complex hydrogel. This hydrogel was then electro-deposited onto a titanium plate with the application of a cathodic voltage, leading to a pH increase and forming a thin opaque surface coat containing IBP-MSN after voltage removal. An in vitro release test revealed the electro-responsive behavior of the system, with the application of a cathodic voltage (−5.0 V) to the titanium plate, leading to a rapid burst release of IBP. Around 95% of the enclosed IBP was released within the first three hours, displaying its potential for controlled drug delivery applications [54].

Recently, Fernandez and his researchers developed conducting polymers with gated MSNs capable of delivering cargo in response to electrochemical stimuli. The MSNs contained surface-modified rhodamine B (Rh B) dye and conductive polymer poly (3,4-ethylene dioxythiophene) (PEDOT) doped with poly [4-styrenesulfonic acid)-co-(maleic acid)]. This functionalization enhanced their optical properties and conductivity, making them potential candidates for applications in imaging and sensing. A bipyridinium derivative was integrated, and heparin (P3) effectively capped the pores through electrostatic interactions. P3 released the confined cargo with the application of a voltage of −640 mV relative to the saturated calomel electrode (SCE). P3 films have biocompatibility with HeLa cells, indicating that they are well-tolerated and compatible with the cells. Additionally, applying a voltage of −600 mV vs. Ag-AgCl triggered the release of rhodamine B from grafted MSN, which was then taken up by HeLa cells [55].

#### 4.1.2. Chemical Stimuli-Responsive MSNs

pH-responsive MSNs: pH plays a vital role in biological systems, including blood circulation and healthy tissues, usually maintained at a steady level of 7.4 [56]. However, several factors can lead to alterations in pH levels. The pH levels in certain cellular compartments, such as cytosols (pH 6.5 to 5.5) and lysosomes (pH 5.5 to 4.5), are notably lower compared to the physiological pH of the body [57]. Researchers have extensively explored the use of pH-responsive strategies to manipulate the behavior and enhance the performance of functionalized MSNs. The development of MSNs can respond to specific pH environments, enabling controlled drug release or targeted delivery of therapeutic agents to acidic cellular regions [41].

Pan et al. (2018) developed a pH-responsive drug delivery system using carboxylated mesoporous silica (COOH-MSN) as a platform. A zeolitic imidazole framework-8 (ZIF-8) film a few nanometers thick was synthesized in situ on the surface of the COOH-MSN. ZIF-8 film acted as a pore blocker, enabling efficient loading of small interfering RNAs (siRNAs) onto the nanoparticles. The remarkably thin ZIF-8 film showcased an exceptional trait related to its capacity to break down within the endo-lysosome’s acidic environment. This served as a trigger for the liberation of enclosed siRNAs and chemotherapeutic agents within the cellular interior. This innovative approach is an advancement, markedly enhancing the efficacy of chemotherapy, especially in the confrontation of drug-resistant cancer cells such as MCF-7/ADR and SKOV-3/ADR cells. The system’s sensitivity to pH fluctuations further contributes to its prowess; it orchestrates a meticulously controlled dispensation of drugs. Specifically, the siRNAs were liberated in response to the distinct pH conditions characteristic of the designated target site [58].

Moorthy et al. (2017) reported synthesized SLN. The surface of nanoparticles was coated with capping units, specifically tetration-maleimide (TTM), to enhance their drug delivery capabilities and responsiveness to changes in pH. The capping units were attached using a “host-guest” complexation mechanism. This modification aimed to create pH-responsive nanoparticles with potential applications in targeting human breast cancer cells, specifically MDA-MB-231 cancer cells. The nanosystem exhibited efficient uptake into the cells, safeguarding the encapsulated cargo molecule (rhodamine B) within its pores and preventing untimely release. This pH-responsive drug delivery system holds significant potential for targeted and controlled release of therapeutic agents, offering promising applications in medicine and pharmaceuticals [59].

Redox-responsive MSNs: Creating redox-responsive vehicles is one of the highly effective approaches for precise and controlled drug delivery, allowing targeted release in response to specific cellular conditions [60]. Redox-responsive drug delivery systems capitalize on the higher concentration of the reducing agent glutathione (GSH) found in tumor cells compared to healthy cells, allowing for targeted drug release in an intracellular environment. This difference enables targeted drug release within tumor cells, enhancing treatment effectiveness while minimizing side effects [61]. Researchers have engineered a pH and redox dual-responsive drug delivery system using MSN-sulfur (SUL)-chitosan (CHS) for controlled release in cancer therapy. This system allows precise drug delivery to cancer cells based on the tumor microenvironment, enhancing treatment efficacy while minimizing off-target effects. The system utilizes an amide reaction to connect dithiodipropionic acid with amino functional groups that can be attached to the surfaces of MSNs and CHS, enabling targeted drug release in response to specific physiological conditions [62]. A model drug, salicylic acid (SA), was used to prepare SA-SUL-MSN-CHS using an impregnation method. The drug loading rate and encapsulation efficiency were calculated as 9.15% and 58.58%, respectively. The in vitro drug release rate was 22.89% under glutathione stimulation, with a significant increase observed as pH decreased [62].

H_2_O_2_-responsive MSNs: Geng and his research group reported the utilization of hydrogen peroxide (H_2_O_2_)-responsive controlled-release nanoparticles for delivering a therapeutic metal chelator in Alzheimer’s disease treatment [63]. The metal chelator’s delivery is achieved with the elevation of H_2_O_2_ levels, showing potential for promising in vivo biomedical applications. This approach holds great prospects for targeted therapies in the future. Xu and his team introduced an innovative microneedle delivery device, which they paired with insulin-loaded and H_2_O_2_-sensitive MSNs for efficient and painless administration. The MSNs nanocarriers were created via surface modification using 4-(Imidazole carbamate) phenylboronic acid pinacol ester (ICBE), and subsequently forming a host–guest complex between ICBE and α-cyclodextrin (α-CD). This approach enhances the functionality of nanocarriers for drug delivery and other biomedical applications. Researchers successfully loaded insulin and glucose oxidase (GOX) into the MSNs. GOX’s enzymatic activity converted glucose into gluconic acid, generating H_2_O_2_ that led to the disintegration of the ICBE-α-CD complex, resulting in the controlled release of insulin. This method enhances the nanocarriers’ capabilities for targeted and controlled drug delivery, holding great promise for various biomedical applications [64].

#### 4.1.3. Biological Stimuli-Responsive MSNs

Enzyme-responsive MSNs: Enzymes acting as triggers in controlled release have sparked significant interest due to their many advantages, including mild reaction conditions, high specificity, and negligible adverse effects. This approach is promising in developing precise and targeted drug delivery systems with improved therapeutic outcomes [65]. A novel nano platform responsive to enzymes has been created to address implant-related bacterial infections and stimulate tissue regeneration in living organisms. In the first step, silver nanoparticles were incorporated into MSNs using a one-pot method. Subsequently, a layer-by-layer assembly approach was applied to create a composite structure, PG-PAH-Ag MSN, sequentially assembling poly-L-glutamic acid (PG) and polyallylamine hydrochloride (PAH) onto Ag-MSN. This process resulted in forming the desired multifunctional composite [66]. The PG-PAH-Ag MSN was applied to polydopamine-modified Ti substrates for surface deposition [63]. In vitro antibacterial experiments demonstrated that Ti substrates coated with a PG-PAH-Ag MSN exhibited outstanding antibacterial efficacy [66]. In a rat model with bacterium-infected femur defects, the modified Ti implants exhibited successful treatment of bacterial infection, as observed in the animal experiment, showcasing their potential for future clinical applications. The results, obtained through micro-CT, hematoxylin-eosin staining, and Masson’s trichrome staining, demonstrated a significant improvement in forming new bone tissue around the modified Ti implants after a 4-week implantation period. These findings suggest the potential of modified implants to enhance bone regeneration and integration in the body. These promising results highlight the potential benefits of the modified implants for bone regeneration applications [66].

ATP-responsive MSNs: Adenosine-5-triphosphate (ATP) serves as the primary energy currency in all living organisms, powering essential biological processes such as muscle contraction, cellular functions, and the synthesis and breakdown of crucial cellular components like DNA, RNA, and histones. Additionally, ATP facilitates membrane transport, ensuring efficient nutrient uptake and waste removal within cells [67,68]. Elevated ATP expression is associated with various pathological processes. Therefore, the demand for ATP-sensitive controlled-release systems in biomedical applications is crucial [69,70]. Zhang et al. (2022) have created a nano platform called MGMs, consisting of metformin (MTF) and glucose oxidase (GOX) co-loaded into manganese silicon nanoparticles (MSNPs). This nano platform delivers dual-inhibited therapy, targeting the tumor microenvironment (TMIE) to starve cancer cells and enhance chemo-dynamic therapy through ATP modulation. In the mildly acidic conditions of the TMIE, the nanoplatforms MGM undergo decomposition, liberating MTF and GOX. This simultaneous release effectively suppresses ATP production by inhibiting oxidative phosphorylation (OXPHOS) and aerobic glycolysis pathways, offering the potential for targeted cancer therapy [71].

Glucose-responsive MSNs: Glucose-sensitive nanomaterials have become a subject of great interest due to their potential to deliver therapeutic agents effectively. Their unique properties enable precise targeting and controlled drug release, making them promising candidates for future medical applications [72]. Zhao and her team developed a glucose-responsive MSN system capable of dual-controlled insulin delivery and cyclic adenosine monophosphate (cAMP). cAMP activates Ca^2+^ channels in pancreas beta cells, rapidly increasing insulin secretion when glucose levels rise. This nano-system shows potential for precise and effective glucose-dependent insulin delivery, offering a promising approach for diabetes management [73]. Qin et al. (2021) developed a novel glucose-responsive oral insulin delivery system utilizing polyelectrolyte complexes (PECs). This system aims to regulate postprandial glucose levels effectively, providing a promising approach for managing blood glucose concentrations after meals. The researchers developed negatively charged alginate-g-3-aminophenylboronic acid (ALG-G-APBA) MSN and positively charged chitosan-g-3-fluoro-4-carboxyphenylboronic acid (CS-G-FPBA) MSN by wrapping ALG-G-APBA and CS-G-FPBA on MSN, respectively. The optimized insulin loading capacities were 128 mg/g for ALG-G-APBA-MSN and 298 mg/g for CS-G-FPBA-MSN, showing their potential as effective drug delivery systems. The PECs exhibited a clear insulin release pattern in a laboratory setting in response to varying glucose concentrations. Notably, the release was regulated to switch “on” during hyperglycemic conditions and “off” during normal glucose levels. A CCK-8 (Cell Counting Kit-8) assay revealed that none of the tested nanoparticles, including MSN, ALG-G-APBA-MSN, CS-G-FPBA-MSN, and PECs, exhibited cytotoxic effects on Caco-2 cells. During in vivo testing, the orally administered PECs demonstrated a notable hypoglycemic effect in diabetic rats, maintaining euglycemic levels for around 12 h. These promising results suggest the potential efficacy of the PECs as a therapeutic approach for managing diabetes [74].

### 4.2. Synthesis/Fabrication Methods

#### 4.2.1. Sol-Gel Method

The cost-effective and straightforward sol-gel method is immensely popular in fabricating MSNs with distinctive surface properties and mesoporous structures. The entire process involves two main stages: hydrolysis and condensation reactions. Specific chemical reactions occur to achieve the desired outcome. Hydrolytic reactions form a colloidal particle solution, which exhibits responsiveness across a broad pH range, including acidic and alkaline conditions. This versatility allows the colloidal particles to be stimulated effectively across a wide spectrum of pH levels.

On the other hand, a condensation reaction takes place at a neutral pH, forming a gel-like structure with three-dimensional networks through crosslinking reactions with siloxane bonds. After the particles undergo a drying procedure, they become receptive to integrating diverse bioactive compounds into the silica gel framework. This unveils intriguing prospects for tailoring nanoparticles to transport distinct therapeutic agents or biomolecules, catering to various medical and biotech applications. Due to the exclusive porous properties and MSNs surface structures, these nanoparticles can effectually control the release of bioactive molecules. This capability enables the preparation of MSNs within a size range of 60–100 nm, offering versatility in various applications [75]. The benefits of this approach are notable. It involves a straightforward 2-step process, saving both time and costs while enabling the production of diverse types of MSNs with precisely controlled mesopore structure and surface properties. Its simplicity and versatility make it attractive for researchers and industries seeking an efficient and customizable synthesis of MSNs for various applications.

Porrang et al. (2021) investigated using natural compounds derived from rice and wheat husks to produce biogenic MSNs through a sol-gel process. These biogenic MSNs loaded with doxorubicin, a chemotherapy drug, demonstrated significant anti-cancer activity against the MCF-7 cell line, indicating their potential as a promising approach for cancer treatment. Using natural compounds to manufacture MSNs highlights an eco-friendly and sustainable method for developing nanocarriers with therapeutic applications [76]. Li et al. (2019) fabricated hollow MSNs using a facile method. They studied the potential of MSNs in improving the solubility, dissolution rate, and bioavailability of Carvedilol (CAR), a poorly water-soluble drug belonging to the Biopharmaceutics Classification System (BSC) type II. Traditional MSNs are commonly prepared using the Stober method, while the fabrication of hollow MSNs with entirely hollow cores involves immersing cetyltrimethylammonium bromide (CTAB) in hot water. The fabricated hollow MSN exhibited remarkable attributes, boasting an expansive surface area measuring 887.76 m^2^/g, a significant pore volume of 0.82 cm^3^/g, and a consistent pore size of 2.19 nm. These characteristics facilitated the effective entrapment of the prototype drug CAR within the hollow MSN structure, thereby amplifying their suitability for various drug delivery implementations. This approach enabled a remarkable drug loading of 41.58  ±  0.56%. In vitro experiments confirmed that CAR-hollow MSN showed a sustained drug release profile compared to pure CAR and CAR-MSN synthesized using the Stober approach. These conclusions revealed the potential of CAR-hollow MSN as a drug delivery system with controlled and prolonged release characteristics. A pharmacokinetic study in rats exhibited a noteworthy enhancement in the bioavailability of CAR. The results recommend that CAR-hollow MSN shows enhanced in vivo absorption and distribution, providing a potential benefit over other drug-delivery formulations [77].

#### 4.2.2. Soft Templating Process

**Figure 4:**
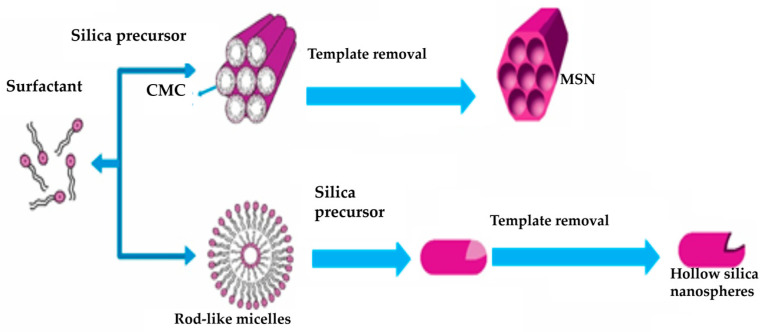
Soft templating process of mesoporous silica nanoparticles preparation. The illustration was reproduced with permission from [78]. CMC: critical micelle concentration; MSN: mesoporous silica nanoparticles.

Preparing hollow MSNs employs a careful and controlled method to produce different, specialized nanostructures. A surfactant template was utilized during this process to create mesoporous cavities within silica nanoparticles, forming hollow structures, as shown in Figure 4.

The ‘soft’ templating method empowers scientists to precisely control factors such as particle dimensions, porosity, and surface characteristics. This approach enables personalized design, proving a versatile and effective way to craft these distinct nanoparticles. These particles hold broad practical utility across various domains, including drug conveyance, catalysis, and sensory applications [79]. The key steps involved in this process are as follows:

Preparation of surfactant solution: a surfactant is combined with a solvent to form a solution. Surfactants consist of components that attract water (hydrophilic) and repel water (hydrophobic).

Formation of surfactant micelles: surfactant molecules autonomously arrange within the solution, creating minuscule micelles with a distinctive core-shell arrangement. The external part of the micelles showcases the hydrophilic ends of the surfactant, while the internal part harbors the hydrophobic tails.

Addition of silica precursors: silica precursors like tetraethyl orthosilicate (TEOS) or tetramethyl orthosilicate (TMOS) are introduced into the solution, encompassing surfactant micelles. These silica precursors then interact with the surfactant template, developing a silica shell around the micelles.

Encapsulation of micelles: the silica building blocks engage with the surfactant micelles, including these micelles within the silica framework.

Hydrolysis and condensation: the initial silica components experience hydrolysis and condensation, yielding silica nanoparticles containing mesoporous cavities that enfold the surfactant micelles. This phase leads to the intended hollow configuration of the nanoparticles. Through skillful management of hydrolysis and condensation reactions, the meticulous control of particle dimensions and porosity becomes feasible, thus establishing an adaptable and promising avenue for fabricating hollow MSNs.

Template removal: this can be attained through solvent extraction or thermal treatment. Once the surfactant template is removed, the desired hollow nanoparticles with well-defined mesoporous structures are achieved [80].

#### 4.2.3. Hard Templating Process

**Figure 5:**
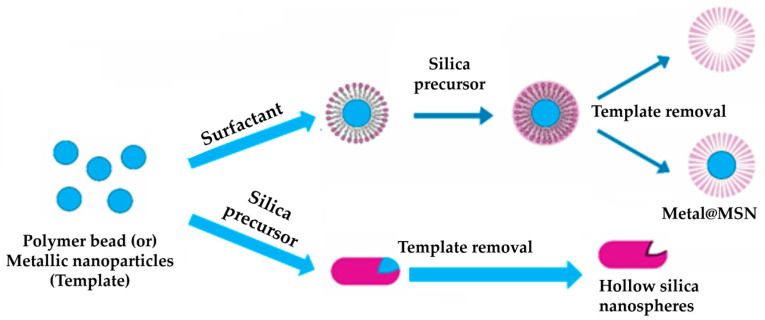
Hard templating process of mesoporous silica nanoparticles (MSNs) preparation. The illustration was reproduced with permission form [78].

The hard templating process is a technique harnessed to produce MSNs. The process involves existing solid nanoparticles or colloids employed as pre-formed hard templates, effectively shaping the desired pore architecture within silica nanoparticles. These hard templates serve as molds, influencing the eventual pore size and arrangement within resultant MSNs. The process commences with the amalgamation of silica precursors like TEOS or TMOS with an appropriate solvent. In this solution, the pre-existing hard templates are introduced and meticulously dispersed. As hydrolysis and condensation reactions transpire, a silica casing evolves around these hard templates, absorbing their structural attributes. Upon the culmination of the condensation process, the hard templates are meticulously extracted from the silica framework, leaving behind a distinct mesoporous configuration. To achieve this, a range of techniques, such as calcination, acid leaching, or solvent extraction, can be harnessed. A visual representation of the hard templating process is depicted in Figure 5.

The precision of the technique offers a straightforward and potent avenue for crafting MSNs endowed with well-defined architectures. This feature renders them immensely valuable across various applications, encompassing drug delivery, catalysis, and sensing [81].

Khoeini et al. (2019) successfully synthesized hollow MSNs using TEOS precursors and a polystyrene template, and the process was carried out in an alcohol-based system with a CTAB surfactant. The synthesized MSN was hollow. Dynamic light scattering analysis and scanning electron microscopy studies confirmed that they were 1 to 10 and 25 to 30 nm in size, respectively [82].

Saputra and colleagues successfully produced hollow MSNs loaded with curcumin (CUR) using the hard templating method. The researchers achieved positive outcomes, particularly enhancing CUR loading onto the nanoparticles. A combination of sonically assisted co-precipitation and L-serine addition was employed to synthesize the hard template, as confirmed through XRD and TEM characterization. This research highlights the effective use of the hard templating approach to create hollow MSNs with enhanced CUR loading potential, presenting opportunities for drug delivery and therapeutic applications [83].

## 5. MSNs for the Treatment of AD

**Figure 6:**
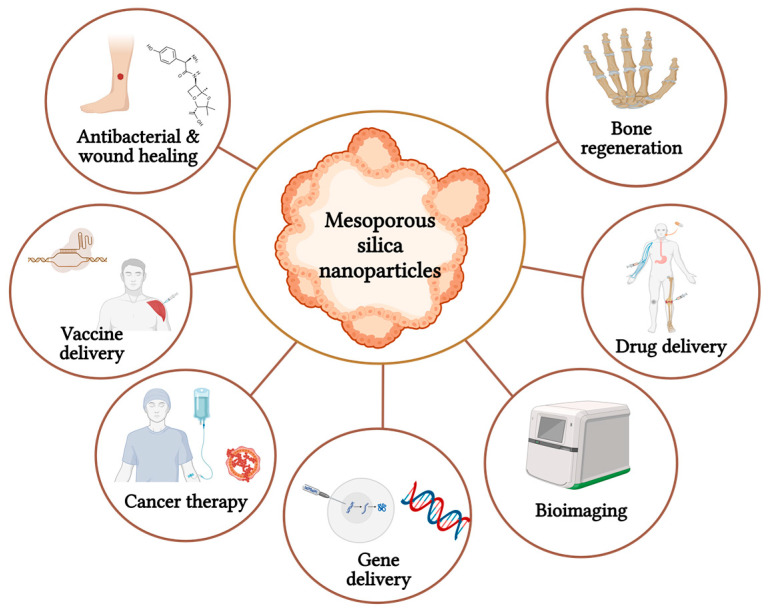
The illustration represents the representative applications of mesoporous silica nanoparticles (created using BioRender.com).

MSNs have garnered significant interest in drug delivery and biomedicine due to their exceptional attributes, such as substantial surface area and extensive pore volume, facilitating effective drug incorporation and precise release mechanisms. This renders them highly promising for pioneering therapeutic uses. Regarding biomedicine applications, MSNs hold immense potential across various domains, encompassing focused drug administration, gene conveyance, and transportation of imaging agents. Due to their distinctive characteristics, they serve as adaptable platforms for therapeutic and diagnostic applications. The adaptability and ability to enhance treatment results have established MSNs as a promising asset in the progression of medical therapies and individualized medicine methodologies [12]. The different biomedical applications of MSNs are shown in Figure 6.

The specific permeability of the BBB poses a significant obstacle in effectively treating neurodegenerative disorders [84]. This protective barrier restricts the passage of many therapeutic agents into the brain, limiting their efficacy in reaching target areas [85]. Overcoming this challenge is crucial for developing successful treatments for such disorders [86].

**Figure 7:**
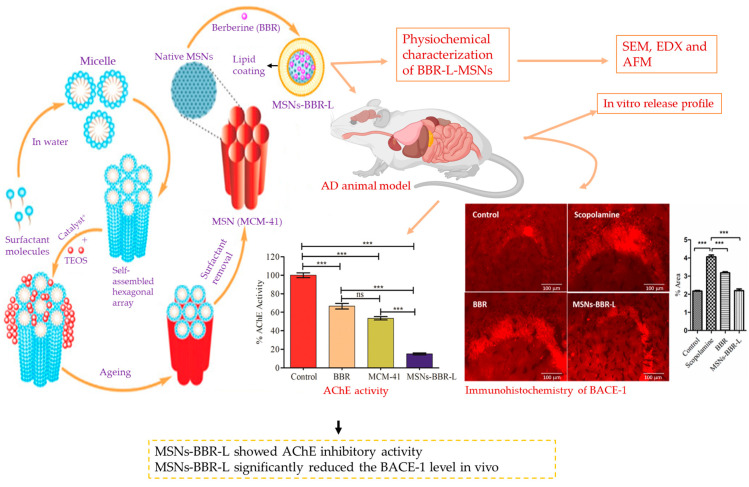
The formulation of lipid-coated MCM-41 MSNs loaded with berberine improved acetylcholine esterase inhibition and amyloid formation. *** Significant difference; ns: no significant; AChE: acetylcholine esterase; TEOS: tetraethylorthosilicate; AD: Alzheimer’s disease; BACE1: beta-site APP-cleaving enzyme 1; AFM: atomic force microscopy; SEM: scanning electron microscopy; EDX: energy-dispersive X-ray spectroscopy (created using BioRender.com). (Recreated with permission from Singh et al., 2021 [87]).

Recently, the development of brain-targeted lipid-coated MSNs loaded with berberine (BB) for AD treatment has been reported. Researchers fabricated the Mobil composition of matter-41 (MCM-41) MSN loaded with BB and coated with lipids (MSNs-BB-L). The lipid coating was achieved using the thin film hydration approach. The size of the synthesized MSNs-BB-L was validated to be between 80 and 100 nm. The MSN-based formulation containing BB-L demonstrated significantly higher acetylcholinesterase (AChE) inhibitory activity than other formulations. The results showed a significant reduction in amyloid fibrillation and malondialdehyde levels with MSNs-BB-L treatment. Both pure BB and MSNs-BB-L treatments led to a notable decline in beta-site amyloid precursor protein cleaving enzyme (BACE-1) compared to scopolamine-intoxicated mice. These outcomes highlight the potential therapeutic benefits of MSNs-BB-L in AD treatment (Figure 7) [87].

**Figure 8:**
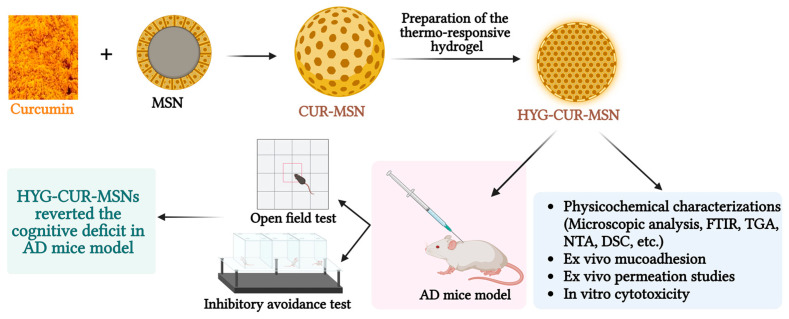
The formulation of MSN loaded with curcumin and HYG-CUR-MSN reversed the cognitive deficit in the AD mice model (Ribeiro et al., 2022). CUR: curcumin; HYG: thermo-responsive hydrogel; AD: Alzheimer’s disease; FTIR: Fourier-transform infrared spectroscopy; TGA: thermogravimetric analysis; NTA: nanoparticle tracking analysis; DSC: differential scanning calorimeter (created using BioRender.com). (Created based on Riberio et al., 2022 [88]).

Riberio et al. (2022) fabricated a CUR-equipped MSN (CUR-MSN), which defeated the limitations (poor water solubility, low bioavailability, and limited efficacy against the primary causes of AD) related to the clinical use of free CUR. By utilizing MSNs, researchers aim to enhance the delivery and effectiveness of CUR in treating AD, thus providing a potential breakthrough in combating this debilitating neurodegenerative condition. A temperature-responsive hydrogel (HYG) presents an intriguing method to facilitate the delivery of the nanosystem through the nasal route and effectively bypass mucociliary clearance mechanisms. The CUR-MSN was incorporated into the hydrogel matrix. Comprehensive physicochemical analyses successfully characterized the properties of the MSNs and CUR-MSN. A remarkable CUR encapsulation efficiency of 88.90 ± 0.04% was reported. Experiments involving CUR-MSN and HYG-CUR-MSN in ex vivo permeation studies demonstrated significant permeation values in porcine nasal mucosa, with 13.12 ± 1.02 μg cm^−2^ of CUR for CUR-MSN and 29.15 ± 1.65 μg cm^−2^ of CUR for HYG-CUR-MSN. Subsequent in vivo investigation conducted in an AD model induced by streptozotocin revealed that HYG-CUR-MSN effectively reversed cognitive deficits in mice. The results highlight the therapeutic potential of HYG-CUR-MSN to treat AD effectively (Figure 8) [88].

**Figure 9:**
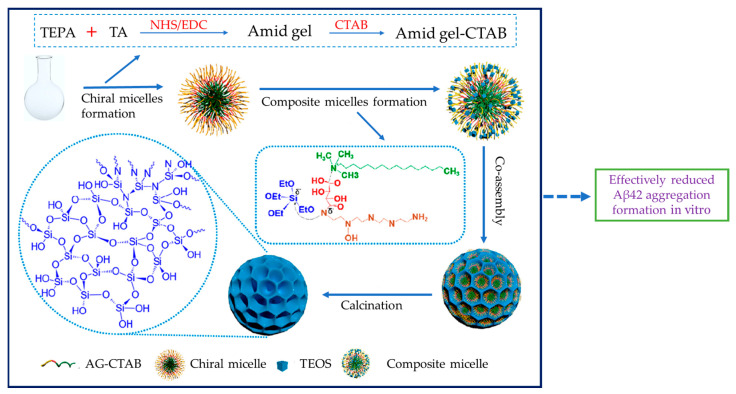
The formulation of chiral skeletons of MSNs and MSNs shows a significant reduction in Aβ42 aggregation. TEPA: tetraethylenepentamine; TA: tartaric acid; AG: amid gel; CTAB: cetyltrimethylammonium bromide; TEOS: tetraethyl orthosilicate; NHS: N-hydroxysuccinimide; EDC: 1-Ethyl-3-(3-dimethylaminopropyl) carbodiimide. (Created using BioRender.com) (recreated with permission from Xu et al., 2023 [89]).

Xu and colleagues reported chiral amide gels-based mSiO_2_ nanospheres. These nanospheres exhibit intricate chiral features at the molecular level within their silicate structures. The inclusion of chiral amide gels through electrostatic interactions induces a chiral attribute in the resulting silica sols. This inventive method enables the creation of specialized chiral nanoparticles with adjustable attributes, holding significant potential for diverse applications like catalysis and nanotechnology. Chiral nanospheres made of mSiO_2_ display a multitude of important mesopores with a diameter of around 10.5 nm. They have substantial pore volumes, 1.6 cm^3^·g^−1^, and showcase significant surface areas, about 528 m^2^·g^−1^. These nanospheres distinctly demonstrate circular dichroism behavior. The chiral frameworks of mSiO_2_ exhibit impressive structural durability, maintaining their chiral characteristics even after high-temperature calcination, including extreme temperatures of 1000 °C. Incorporating chiral mSiO_2_ has shown a substantial decrease, up to 80%, in the formation of aggregates of β-amyloid protein (Aβ42). This decline in aggregation significantly curbs Aβ42-induced cytotoxic effects on human neuroblastoma cells (SH-SY5Y) (Figure 9) [89].

**Figure 10:**
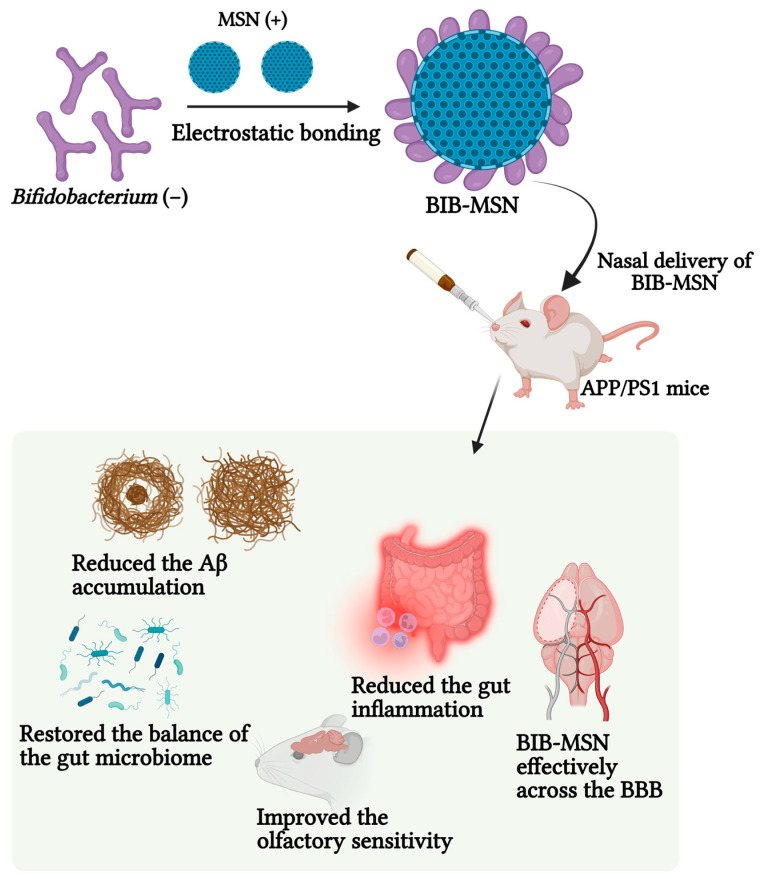
The formulation of Bifidobacterium-MSN (BIB-MSN) and BIB-MSN improved the gut microbiome and olfactory sensitivity and reduced gut inflammation and Aβ accumulation in APP/PS1 mice. BBB: blood–brain barrier; Aβ: amyloid beta. (Created using BioRender.com) (created based on Liu et al., 2022 [90]).

Another study reported the feasibility of intranasally delivering Bifidobacterium-MSN (BIB-MSN) to the gut. The integration of MSNs nanospheres onto BIB’s surface was achieved successfully. Upon intranasal administration, BIB-MSN showcased effective transportation across the BBB to the peripheral intestine, substantiated with fluorescence imaging within the abdominal and gastrointestinal regions. This method shows exciting prospects for future therapeutic uses, capitalizing on BIB’s transport abilities and MSNs’ drug delivery capacity to address specific body areas accurately. The intranasal administration of BIB-MSN exhibited positive outcomes in APP/PS1 mice, diminishing gut inflammation and decreasing cerebral Aβ buildup and heightening the sense of smell. These results highlight the potential benefits of BIB-MSN in managing AD. The findings suggest that restoring balance in the gut microbiome is vital in easing cognitive decline in AD. Administering BIB-MSN through the nose is a promising treatment method for preventing AD and managing intestinal ailments. These revelations underscore the relevance of interactions between the gut and brain in conditions like neurodegenerative diseases, paving the way for an innovative and personalized approach to tackling AD and associated gastrointestinal issues (Figure 10) [90]. The representative studies of drugs-loaded MSNs and their characterization have been listed in Table 1.

## 6. Representative Applications of MSNs in the Medical Sector

MSNs have diverse biomedical applications, including antitumor therapy, addressing bone disorders, and combating infectious diseases. Furthermore, MSNs are effective carriers for dyes and contrast agents, contributing to their diagnostic utility in medical imaging and research. The distinctive attributes of MSNs have extended their utility from drug delivery to the delivery of nucleic acids, including miRNA, in biomedical applications. MSNs have successfully facilitated the delivery of drugs like anticancer and anti-inflammatory agents while also serving as carriers for immune-enhancing compounds. Some of the applications of MSNs are detailed below.

### 6.1. Wound Healing and Tissue Regeneration

MSNs have appeared as promising candidates for wound healing and the regeneration of tissues [94]. These nanoparticles offer an extremely porous structure, allowing for effectual encapsulation and controlled release of therapeutic medicaments, which can enhance the healing process and encourage tissue regeneration. Their surface can be readily tailored with specific ligands or growth factors, enabling targeted delivery to the wound site [95].

Wu et al. (2018) developed ROS-responsive functionalized MSNs as nanocarriers to facilitate wound healing. These nanocarriers are responsive to ROS present at the wound site. This responsiveness permits them to release therapeutic agents exactly where ROS levels surge, thus escalating the wound-healing process. An innovative nanocomposite was fabricated by combining minute ceria nanocrystals onto the exterior of amine-modified MSN structures. Incorporating ceria nanocrystals promoted strong tissue adhesion and enhanced wound-healing, demonstrating its dual advantages in the system. In addition, it led to greater development of skin appendages and reduced scar formation, contributing to overall healing outcomes. The blend of ceria nanocrystals and amino-functionalized MSNs offers a productive solution for addressing oxidative stress-related issues and encouraging tissue repair and regeneration [94].

The potential of positively charged functionalized MSNs as nanocarriers for delivering hepatocyte nuclear factor 3β plasmid DNA (pHNF3β) has been reported. The study showed the improved differentiation of induced pluripotent stem cells into hepatocyte-like cells within a short period of in vitro culture. Enhancing the delivery frequency of pHNF3β led to a more pronounced improvement in differentiation. Utilizing functionalized MSN as nanocarriers enables precise and targeted delivery of pHNF3β, promoting the intended cellular differentiation mechanism. The study showed therapeutic advancement in liver regeneration and tissue engineering [96].

Studies have demonstrated that MSNs modified with PEI are efficient carriers for growth factors, directing the development of mouse embryonic stem cells (mESCs) into hepatocyte-like cells in vitro and in vivo. The results indicate that MSNs show promise in regenerative medicine and tissue engineering. The developed system verified advancements in differentiating cells towards induced hepatocyte-like cells (iHeps) in vitro. iHeps showed mature functions, signifying their potential for therapeutic applications. The system efficiently encouraged the regeneration of damaged hepatic tissues after four weeks when transplanted into mice with liver injury. The results emphasize the system’s potential in vitro cell differentiation and in vivo tissue regeneration, offering a promising path for upcoming biomedical interventions. The modified MSNs effectively transport growth factors for directing stem cell differentiation, paving the route for potential use in liver regeneration and additional tissue healing treatments [97].

### 6.2. Imaging and Diagnostic Application

Magnetic nanoparticles have been integrated into MSN structures, adding magnetic attributes that enable the manipulation of payload discharge or functioning as agents for magnetic resonance imaging (MRI) enhancement. This modification can precisely govern drug release by applying external magnetic fields, thus amplifying the precision of targeted therapeutic interventions. Among the magnetic nanoparticles utilized in biomedical research, two popular choices are Fe_3_O_4_ and Fe_2_O_3_. These magnetic nanoparticles are usually used to functionalize MSN and serve as MRI-sensitive probes. By integrating magnetic nanoparticles into MSNs, scientists can craft versatile nanocarriers. These structures not only aid in drug transport and gene therapy but also provide the capability for non-intrusive MRI imaging [98,99].

### 6.3. Biocatalyst

MSNs hold incomparable properties, rendering them well-suited for different biocatalytic applications. The distinctive attributes of MSNs are suitable for effective enzyme immobilization, precise drug administration, and improved treatment outcomes, positioning them as a promising avenue for advancing medical applications and catalytic processes. Their significant surface area and distinguished absorption capabilities streamline the loading and conveyance of enzymes, thus optimizing catalytic reactions. MSNs offer outstanding mechanical stability and consistent pore distribution, offering a stable and controlled environment for encapsulating enzymes, shielding them from proteolysis, and extending their enzymatic activity. MSNs’ functionalizable surface enables easy modification, facilitating targeted delivery and reducing immunological responses during intercellular bioanalysis. MSNs’ characteristics make them highly compatible with encapsulating and safeguarding enzymes, thereby improving the resistance and effectiveness of enzymes in a range of biological applications [100,101].

A novel nanosystem has been fabricated to evaluate tumor development employing self-catalyst luminescence. This nanosystem consists of an MSN loaded with luciferin, a bioluminescent compound. As a capping piece, the MSN is functionalized with AuNPs, connected through disulfide bonds. The nanosystem is conjugated with PEGylated luciferase, an enzyme that raises the luminescence signal. The luciferin acts as the substrate for luciferase in this fabricated nanosystem. When the nanosystem encounters a tumor microenvironment at a specific pH or elevated levels of certain enzymes, the disulfide bonds are cleaved, facilitating the release of luciferin from the MSN. The luciferin engages with PEGylated luciferase, leading to detectable luminescence production. This self-sustaining luminescence acts as a catalyst, enabling live tracking of tumor growth without requiring external luciferin addition. Incorporating MSNs, AuNPs, and PEGylated luciferase provides benefits such as increased stability, biocompatibility, and improved tumor-homing capabilities [102].

Another study reported the fabrication of a nanosystem through the on-site formation of AuNPs on the surface of an amino-functionalized MSN. The nanosystem established notable nanoreactor capabilities with enzyme-mimicking catalytic properties. It competently facilitated a cascade reaction through self-activation, showcasing its potential for diverse applications. The nanosystem replicated the function of GOX by catalyzing the oxidation of glucose using oxygen (O_2_), resulting in the production of gluconic acid and H_2_O_2_. This enzyme-like performance of the nanosystem paves the way for various biomedical and environmental uses, capitalizing on the effective catalytic characteristics of AuNPs present on the MSN surface. This arrangement allows the design of artificial enzymes with various capabilities and chemical responses, showcasing the potential for versatile applications [103].

## 7. Advantages and Limitations

A notable benefit of MSNs is their extensive surface area due to their porous structure. This distinctive characteristic provides abundant room for drug encapsulation and functional molecule binding, rendering them highly valuable in medical contexts. The substantial surface area of MSNs enables improved drug administration tailored to specific treatments, establishing their significance in the medical realm. Additionally, MSNs can regulate and gradually discharge drugs, heightening treatment effectiveness and reducing the likelihood of undesirable repercussions [104]. This controlled release allows for a more targeted and productive delivery of therapeutic agents, contributing to enhanced patient outcomes and a more patient-friendly treatment experience. Biocompatibility is a vital advantage of MSNs, making them compatible with safe medical applications. Researchers can surge their biocompatibility through further alterations, guaranteeing their compatibility with the human body and promoting their use in several medical treatments and therapies [105].

MSNs exhibit distinct advantages for applications in AD research and therapy. Bare MSNs, characterized by a large surface area, offer efficient drug loading and controlled release capabilities, making them ideal for drug delivery systems in AD treatment [63]. Surface-modified MSNs take these advantages a step further, allowing for targeted drug delivery to specific regions in the brain affected by AD and controlled release to minimize side effects. MSNs with integrated imaging agents, such as fluorescent dyes or contrast agents, are crucial in AD diagnosis and monitoring, aiding in early detection and disease progression tracking [106]. MSNs designed for gene therapy enable the delivery of therapeutic genes, addressing genetic aspects of AD treatment [107]. Dual-functional MSNs, combining drug delivery with imaging, provide a multimodal approach, offering diagnostic and therapeutic benefits in a single platform [108]. Chitosan-coated MSNs enhance biocompatibility and enable sustained drug release, making them valuable in managing AD symptoms over extended periods [109]. The diverse features of MSNs, from drug delivery and targeting to imaging and gene therapy, promise innovative and multifaceted strategies for tackling AD, offering potential advancements in diagnosis, treatment, and patient care.

The surface of MSNs can be tailored with dissimilar molecules, making them extremely adaptable for specific purposes like targeted drug delivery and interactions with specific cells in the body. This flexibility allows for tailoring MSNs to suit the unique needs of several medical applications, increasing their effectiveness in delivering therapies exactly where they are needed [78]. MSNs also serve as versatile carriers for drugs and biomolecules such as DNA, RNA, and proteins. This exclusive capability of MSNs enables their application in gene therapy and several other biomedical uses, offering important solutions for targeted treatments and advanced medical interventions [110].

**Figure 11:**
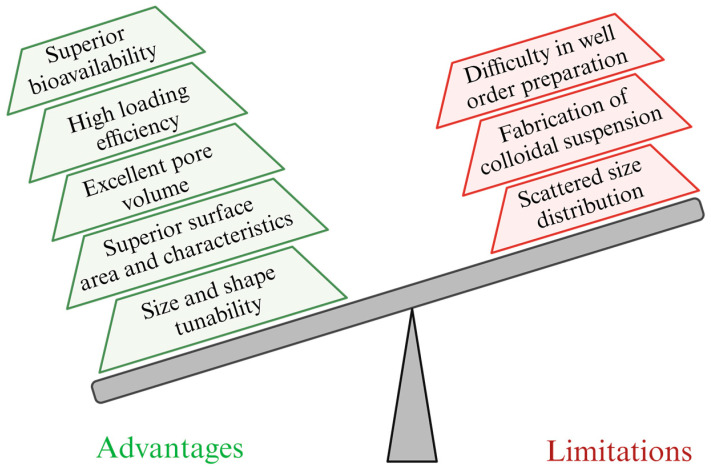
The advantages and limitations of MSNs.

MSNs show low immunogenicity, which means they are less likely to provoke immune responses when administered in the body. These characteristics augment their safety and lessen the risk of adverse reactions, making them a promising alternative for biomedical applications. MSNs exhibit minimal immunogenicity, reducing the likelihood of provoking immune reactions upon administration. These adaptable nanoparticles can act as contrast agents in medical imaging, including MRI and fluorescence imaging, to diagnose specific diseases [111]. These features make MSNs valuable tools in medical applications, ensuring safer drug delivery and precise disease detection and monitoring. The unique blend of drug delivery and imaging capabilities in MSNs makes them well-suited for theranostic applications. This allows for both administering therapy and monitoring treatment’s effectiveness simultaneously. Such versatility in one nanoparticle system holds great promise in advancing personalized and effective medical interventions for patients [112]. Figure 11 shows the advantages and limitations of multifunctional MSNs.

The difficulty in preparing MSNs can be solved by introducing more intricate structural features and greater control over particle size and pore size distribution. This may entail employing novel, complex templates or surface modification techniques to create intricate pore structures, which can be particularly challenging to design and replicate. Additionally, achieving a high degree of uniformity and homogeneity in particle size while maintaining a mesoporous structure can elevate the difficulty, requiring precise control over synthesis conditions and meticulous characterization to ensure the desired product quality. Such advancements contribute to the evolving field of nanotechnology while posing increased technical challenges to researchers in pursuing tailored MSNs.

Several strategies can be employed to enhance the scattered size distribution of MSNs. Firstly, rigorous control over synthesis parameters, such as reactant concentrations, reaction time, and temperature, is crucial to achieve uniform particle sizes. Utilizing a steric stabilizer or surfactant during the synthesis can help prevent particle aggregation and improve size homogeneity. Furthermore, post-synthesis techniques like size-selective centrifugation or filtration can effectively isolate nanoparticles within the desired size range [113]. Finally, regular characterization and optimization using techniques like dynamic light scattering can help monitor and fine-tune size distribution during production, ensuring consistent and improved quality of MSNs.

MSNs have demonstrated promising possibilities in an extensive range of applications. However, it is essential to acknowledge that they come with certain limitations, including reduced drug encapsulation efficacy, deprived compatibility, and poor degradability, resulting in poor therapeutic outcomes [114] that require careful consideration. These factors might influence their optimal use and safety in different contexts. To harness the full potential of MSNs, researchers actively address these challenges through improved design and continued investigation, aiming to overcome any hurdles that might hinder their effective and safe application in diverse fields [115]. Although MSNs are commonly regarded as biocompatible, it is essential to be cautious about potential immune responses or toxicity concerns arising from specific surface modifications or impurities. The rate at which MSNs biodegrade can differ based on size, shape, and surface properties. Extended retention of undegraded MSNs in the body may lead to safety concerns that need careful consideration.

Cargo leakage is one of the limitations of MSNs, which are used in drug delivery, where the therapeutic payload inside the nanoparticles may be released prematurely before reaching the intended target site [116], resulting in a decrease in the effectiveness of the treatment and hinder its ability to achieve the desired therapeutic outcome.

How cells take up MSNs is not completely understood, and the efficiency of uptake can differ based on the type of cell and the surface properties of the nanoparticles. Off-target effects can occur with MSNs, where these nanoparticles may unintentionally accumulate in tissues or organs other than the intended target site, potentially causing undesired side effects [117]. Ensuring the precise delivery and localization of MSNs to the intended areas remains an important consideration to minimize such off-target effects and enhance the safety and efficacy of their applications. The immune system can identify and remove MSNs, reducing their circulation time and potentially impacting their effectiveness [118]. This immune recognition may influence their behavior in the body and affect their overall performance as drug-delivery vehicles or therapeutic agents. One of the limitations of MSN is the difficulty in scaling up their production consistently and uniformly. This challenge hinders their widespread practical applications and requires further attention to enable their efficient large-scale manufacturing for various biomedical purposes [119]. The safety of using MSNs in the long term and the possibility of their accumulation in organs over time require additional in-depth research and investigation. We need a better understanding of how these nanoparticles interact with the body over extended periods to ensure their safe and effective use in various applications. Obtaining regulatory approval for using MSNs in clinical applications can be intricate and time-consuming, requiring rigorous evaluation and adherence to strict guidelines and safety standards [120].

## 8. Future Perspective

Preclinical and clinical studies on MSNs have demonstrated their significant advantages as drug carriers in drug delivery systems. These MSNs hold enormous potential for treating diverse diseases due to their beneficial effects in effectively delivering therapeutic agents. Despite frequent clinical studies on MSNs, factors must be addressed before their widespread commercial application. It is essential to conduct rigorous in vitro and in vivo investigations to advance MSNs-based formulations toward clinical evaluation. These investigations should address acute to long-term toxicity, immunity, and biodistribution concerns to ensure their safety and efficacy in human trials. Also, by refining the methods and processes, MSNs can be employed more effectively to address several medical challenges and augment drug delivery and therapeutic outcomes.

## 9. Conclusions

AD treatment faces significant challenges, with the accumulation of Aβ peptides in the brain being a hallmark. The potential of MSNs has been broadly explored for delivering drugs to treat AD. These minute silica particles possess distinctive attributes, including a substantial surface area relative to their dimensions, the potential for tailoring pore size, and a notable capability to efficiently traverse the BBB. This makes them a promising alternative for delivering therapeutic agents to the brain with superior precision and effectiveness. It has been reported that MSNs possess noteworthy properties as nano scavengers, making them a promising candidate to treat neurodegenerative disorders. The accumulation of harmful substances like Aβ peptides in the brain characterizes AD. These ingenious nanoparticles can effectively bind to and eliminate these toxic molecules from the brain, offering new hope in the battle against neurodegenerative diseases. The potential therapeutic application of these nanoparticles opens exciting possibilities for tackling these challenging conditions and improving patients’ lives. MSNs also showed excellent potential as nanocarriers for wound healing and tissue regeneration. They also exhibited noteworthy applications in imaging and diagnostics. Despite the influential role of MSNs in biomedical applications, there remains a crucial need for extensive research to understand the safety implications associated with engineered nanoparticles. A cautious approach is essential in evaluating MSNs before they are applicable in the clinical sector.

## Figures and Tables

**Table 1:** The representative drug-loaded MSNs and their characterization.

MSNs/ Subtype	Drug Used	Method of Preparation	Physiochemical Parameters	Outcomes	Ref.
MCM-41	BBR	Passive approach	Entrapment efficiency: 75.21 ± 1.59% Particle size: 143 ± 11 nm Polydispersity index: 0.30 ± 1.28 Zeta potential: 8.7 ± 1.9 mV	MSNs-BBR-L showed AChE inhibitory activity. MSNs-BBR-L significantly reduced the BACE-1 level in vivo.	[87]
MCM and SBA, PHTS, MCF	L-Dopa	Soft templating approach	MCM-41(S): Particle size: 200 to 900 nm Surface area: 880 m^2^/g Pore volume: 0.72 cm^3^/g Loading capacity: 5.1 ± 0.3% MCM-41(HO): Particle size: 400 to 600 nm Surface area: 1120 m^2^/g Pore volume: 0.75 cm^3^/g Loading capacity: 5.3 ± 0.5% MCM-48: Particle size: >1000 nm Surface area: 470 m^2^/g Pore volume: 0.77 cm^3^/g Loading capacity: 4.9 ± 0.3% PHTS: Particle size: >1000 nm Surface area: 940 m^2^/g Pore volume: 0.83 cm^3^/g Loading capacity: 4.2 ± 0.6% MCF: Particle size: >1000 nm Surface area: 760 m^2^/g Pore volume: 1.88 cm^3^/g Loading capacity: 5.6 ± 0.2% SBA-15: Particle size: 300 to 500 nm Surface area: 1020 m^2^/g Pore volume: 1.17 cm^3^/g Loading capacity: 5.9 ± 0.3%	SBA-15 with elevated surface area and substantial pore volume was developed. SBA-15 exhibited the most significant drug release.	[91]
MCM-41	Rivastigmine	Solvent equilibrium approach	Entrapment efficiency: 89 ± 1.45% Particle size: 145 ± 0.5 nm Zeta potential: −38.9 ± 1.4 mV	Delivered rivastigmine (127 fold) effectively to the brain in vivo. The bioavailability of the drug was increased (12.3 fold). The efficiency of encapsulated drugs was higher than naked drugs. Choline-esterase and amyloid formation inhibition activity.	[92]
--	Quercetin	Sol-gel approach	Entrapment efficiency: 71.65% Pore size: 169.97 Å Pore volume: 0.75 cm^3^/g Surface charge −34 ± 0.55 mV Surface area: 109.34 m^2^/g	Drug-loaded nanocarriers showed enhanced stability and solubility of the drug. Anti-amyloid and antioxidant activity.	[93]

MSN: mesoporous silica nanoparticles; BBR: berberine; SBA: Santa Barbara amorphous; MCM: Mobil composition of matter; HO: highly ordered; S: spherical; PHTS: plugged hexagonal templated silica; MCF: mesostructured cellular foam; MSNs-BBR-L: MSN-loaded with berberine; BACE1: beta-site APP-cleaving enzyme 1.

## Data Availability

No new data was created in this study. Data sharing does not apply to this article.
